# Supplementary material for: Statistical Analysis of Common Respiratory Viruses Reveals the Binary of Virus-Virus Interaction
Source: Microbiol Spectr. 2023 Jun 28;11(4):e00019-23. doi: 10.1128/spectrum.00019-23 (PMC10433823; doi:10.1128/spectrum.00019-23)
Supplement: Supplemental file 1 — Supplemental material. Download spectrum.00019-23-s0001.pdf, PDF file, 0.6 MB [file spectrum.00019-23-s0001.pdf]

Supplementary materials

Supplement to: Statistical analysis of common respiratory viruses reveals the binary of virus-virus interaction

Table S1. Characteristics of patients suffered from acute respiratory tract infections in Beijing, China, 2005-2015.

Characteristics	All patients (n=14,426)	Age groups				*P*-value
		14-24 yrs (n=3,932)	25-44 yrs (n=6,835)	45-64 yrs (n=2,649)	65+ yrs (n=963)	
Female gender, no. (%)	7818 (54.19)	2066 (52.54)	3770 (55.16)	1505 (56.81)	454 (47.14)	<0.001
Pregnant, no. (%)	70/7818 (0.90)	11/2066 (0.53)	59/3770 (1.56)	0 (0)	0 (0)	<0.001
Underlying diseases, no. (%)	954 (6.61)	75 (1.91)	245 (3.58)	366 (13.82)	267 (27.73)	<0.001
Hypertension [a]	306 (2.12)	3 (0.08)	39 (0.57)	139 (5.25)	125 (12.98)	<0.001
Chronic liver, heart or renal disease [b]	196 (1.36)	7 (0.18)	31 (0.45)	66 (2.49)	92 (9.55)	<0.001
Diabetes	144 (1.00)	3 (0.08)	18 (0.26)	55 (2.08)	67 (6.96)	<0.001
Cancer	133 (0.92)	7 (0.18)	33 (0.48)	58 (2.19)	35 (3.63)	<0.001
Chronic lung disease [c]	83 (0.58)	9 (0.23)	17 (0.25)	25 (0.94)	32 (3.32)	<0.001
Primary diagnosis, no. (%)						
URTI [d]	14016 (97.16)	3876 (98.58)	6686 (97.82)	2554 (96.41)	853 (88.58)	<0.001
LRTI [e]	410 (2.84)	56 (1.42)	149 (2.18)	95 (3.59)	110 (11.42)	<0.001
Antibiotics usage before visiting, no. (%)	2082 (14.43)	551 (14.01)	993 (14.53)	434 (16.38)	98 (10.18)	<0.001
β-Lactam alone	1296 (8.98)	359 (9.13)	617 (9.03)	266 (10.04)	52 (5.40)	<0.001
Macrolide alone	482 (3.34)	125 (3.18)	238 (3.48)	94 (3.55)	23 (2.39)	0.284

Fluoroquinolone alone	276 (1.91)	54 (1.37)	138 (2.02)	65 (2.45)	18 (1.87)	0.014
β-Lactam plus Macrolide	26 (0.18)	9 (0.23)	12 (0.18)	4 (0.15)	1 (0.10)	0.811
Anti-viral usage before visiting, no. (%)	26 (0.18)	8 (0.20)	13 (0.19)	3 (0.11)	2 (0.21)	0.837

[a] Hypertension, classified when hypertension (ICD-9: 401.xx International Classification of Diseases, ninth revision) was diagnosed or patient was on the medication of anti-hypertensive medications during visit.

[b] Chronic liver, heart or renal diseases, classified when liver cirrhosis, chronic hepatitis, liver failure, alcoholic liver disease, ischemic heart disease, rheumatic heart disease, chronic kidney diseases or renal failure (ICD-9: 393-398, 410-414, 571, 585-586.xx) were diagnosed during visit.

[c] Chronic lung diseases, defined as emphysema, chronic bronchitis, asthma, bronchiectasis or chronic airway obstruction (ICD-9: 490-494, 496.xx).

[d] URTI=upper respiratory tract infection, classified when common cold, acute rhinitis, pharyngitis, sinusitis, tonsillitis, laryngitis or acute otitis media (ICD-9: 460-465.xx International Classification of Diseases, ninth revision) were diagnosed during visit.

[e] LRTI=lower respiratory tract infection classified when acute bronchitis, bronchiolitis, pneumonia and tracheitis (ICD-9: 466, 480-486.xx) were diagnosed during visit.

Table S2. Frequency of respiratory viruses in adult suffered from acute respiratory tract infections by age group in Beijing, China, 2005-2015.

Parameters	Total tested (%) (n=14,426)	Age groups				*P*-value
		14-24 yrs (n=3,932)	25-44 yrs (n=6,835)	45-64 yrs (n=2,649)	65+ yrs (n=963)	
No. of positive detections	5585 (38.71)[a]	1600 (40.69)	2582 (37.78)	1020 (38.51)	363 (37.69)	0.023
Single-detection	5243 (36.34)	1494 (38.00)	2421 (35.42)	968 (36.54)	341 (35.41)	0.056
Influenza viruses	3238 (22.45)	851 (21.64)	1545 (22.60)	644 (24.31)	189 (19.63)	0.01
A	2399 (16.63)	631 (16.05)	1151 (16.84)	460 (17.37)	151 (15.68)	0.418
B	814 (5.64)	210 (5.34)	383 (5.60)	181 (6.83)	37 (3.84)	0.003
C	25 (0.17)	10 (0.25)	11 (0.16)	3 (0.11)	1 (0.10)	0.5
Human rhinoviruses	798 (5.53)	273 (6.94)	360 (5.27)	114 (4.30)	47 (4.88)	<0.001
Human parainfluenza viruses	385 (2.67)	107 (2.72)	146 (2.14)	89 (3.36)	42 (4.36)	<0.001
1	101 (0.70)	30 (0.76)	40 (0.59)	22 (0.83)	8 (0.83)	0.491
2	73 (0.51)	31 (0.79)	31 (0.45)	8 (0.30)	3 (0.31)	0.024
3	139 (0.96)	26 (0.66)	48 (0.70)	43 (1.62)	22 (2.28)	<0.001
4	72 (0.50)	20 (0.51)	27 (0.40)	16 (0.60)	9 (0.93)	0.125
Enteroviruses	355 (2.46)	137 (3.48)	185 (2.71)	22 (0.83)	9 (0.93)	<0.001
Human adenoviruses	151 (1.05)	68 (1.73)	69 (1.01)	12 (0.45)	1 (0.10)	<0.001
Human coronaviruses	129 (0.89)	37 (0.94)	36 (0.53)	28 (1.06)	27 (2.80)	<0.001
NL63	11 (0.08)	6 (0.15)	2 (0.03)	1 (0.04)	2 (0.21)	0.054
HKU1	14 (0.10)	3 (0.08)	6 (0.09)	2 (0.08)	3 (0.31)	0.178
OC43	75 (0.52)	17 (0.43)	22 (0.32)	18 (0.68)	18 (1.87)	<0.001
229E	23 (0.16)	8 (0.20)	6 (0.09)	7 (0.26)	1 (0.10)	0.183
Respiratory syncytial virus	98 (0.68)	6 (0.15)	44 (0.64)	30 (1.13)	18 (1.87)	<0.001
Subgroup A	63 (0.44)	4 (0.10)	30 (0.44)	18 (0.68)	11 (1.14)	<0.001
Subgroup B	35 (0.24)	2 (0.05)	14 (0.20)	12 (0.45)	7 (0.73)	<0.001
Human metapneumovirus	84 (0.58)	14 (0.36)	32 (0.47)	27 (1.02)	10 (1.04)	0.001
Human bocaviruses	11 (0.08)	4 (0.10)	4 (0.06)	2 (0.08)	1 (0.10)	0.871
Co-detection	342 (2.37)	106 (2.70)	161 (2.36)	52 (1.96)	22 (2.28)	0.292
Two-pathogen	322 (2.23)	98 (2.49)	153 (2.24)	49 (1.85)	21 (2.18)	-
Three-pathogen	18 (0.12)	7 (0.18)	8 (0.12)	2 (0.08)	1 (0.10)	-
Four-pathogen	2 (0.01)	1 (0.03)	0 (0)	1 (0.04)	0 (0)	-

[a] Numbers in parentheses are percentages.

Table S3. The Frequency of dual-detected pathogens in patients suffered from acute respiratory tract infections.

Co-detected virus	IFV-A	IFV-B	IFV-C	HRVs	HPIV-1	HPIV-2	HPIV-3	HPIV-4	EVs	Adv	HKU1	OC43	229E	NL63	RSV-A	RSV-B	hMPV	HBoV	Total
IFV-A	-	0	10	83	4	3	5	3	18	11	1	3	3	2	8	5	6	8	165
IFV-B	0	-	3	34	1	1	1	3	6	6	0	1	3	0	6	6	5	0	73
IFV-C	10	3	-	3	0	0	0	0	1	1	0	0	0	0	0	1	0	1	16
HRVs	83	34	3	-	4	2	6	1	29	13	1	3	2	2	2	5	1	0	173
HPIV-1	4	1	0	4	-	0	4	0	4	1	0	0	0	0	0	0	1	0	17
HPIV-2	3	1	0	2	0	-	2	2	1	0	0	0	2	1	0	1	0	0	14
HPIV-3	5	1	0	6	4	2	-	3	4	2	2	1	0	0	1	1	0	0	28
HPIV-4	3	3	0	1	0	2	3	-	3	0	0	0	0	0	0	0	0	1	14
EVs	18	6	1	29	4	1	4	3	-	3	0	4	1	1	0	3	1	1	65
Adv	11	6	1	13	1	0	2	0	3	-	0	0	2	0	2	1	1	0	38
HKU1	1	0	0	1	0	0	2	0	0	0	-	0	0	0	0	0	0	0	4
OC43	3	1	0	3	0	0	1	0	4	0	0	-	0	0	1	0	1	0	14
229E	3	3	0	2	0	2	0	0	1	2	0	0	-	0	0	0	0	0	12
NL63	2	0	0	2	0	1	0	0	1	0	0	0	0	-	0	0	0	0	6
RSV-A	8	6	0	2	0	0	1	0	0	2	0	1	0	0	-	0	2	0	21
RSV-B	5	6	1	5	0	1	1	0	3	1	0	0	0	0	0	-	0	0	20
hMPV	6	5	0	1	1	0	0	0	1	1	0	1	0	0	2	0	-	0	17
HBoV	8	0	1	0	0	0	0	1	1	0	0	0	0	0	0	0	0	-	9

Table S4. Correlation matrix of residuals obtained by vector autoregressive model.

	Correlation matrix of residuals									
	IFV-A	IFV-B	HPIV 2/4	HPIV 1/3	HCOVs-β	Adv	HCOVs-α	hMPV	RSV	picoRNA
IFV-A	1.000									
IFV-B	-0.150	1.000								
HPIV 2/4	-0.037	-0.105	1.000							
HPIV 1/3	-0.116	0.000	0.259	1.000						
HCOVs-β	-0.031	-0.058	0.102	-0.168	1.000					
Adv	0.014	-0.188	0.121	0.208	-0.066	1.000				
HCOVs-α	-0.026	0.025	-0.101	0.110	-0.208	0.174	1.000			
hMPV	-0.123	0.036	0.011	0.004	0.115	0.269	0.042	1.000		
RSV	0.047	0.360	-0.169	-0.203	-0.048	0.017	-0.110	0.057	1.000	
picoRNA	-0.055	-0.157	0.123	0.177	0.013	0.001	-0.110	-0.055	-0.118	1.000

HPIV 1/3 = human parainfluenza virus (1 and 3); HPIV 2/4= human parainfluenza virus (2 and 4); HCoVs-α = human coronaviruses (229E, NL63); HCoVs-β = human coronaviruses (OC43, HKU1); picoRNA = picornaviridae virus (human rhinoviruses, enteroviruses).

Table S5. Covariance matrix of residuals obtained by vector autoregressive model.

Covariance matrix of residuals										
	IFV-A	IFV-B	HPIV 2/4	HPIV 1/3	HCOVs-β	Adv	HCOVs-α	hMPV	RSV	picoRNA
IFV-A	5.02E-02									
IFV-B	-3.62E-03	1.17E-02								
HPIV 2/4	-1.54E-04	-2.10E-04	3.42E-04							
HPIV 1/3	-3.86E-04	-4.80E-07	7.10E-05	2.20E-04						
HCOVs-β	-1.50E-04	-1.36E-04	4.12E-05	-5.46E-05	4.78E-04					
Adv	5.32E-05	-3.54E-04	3.92E-05	5.38E-05	-2.51E-05	3.05E-04				
HCOVs-α	-7.72E-05	3.63E-05	-2.51E-05	2.19E-05	-6.09E-05	4.07E-05	1.79E-04			
hMPV	-2.96E-04	4.22E-05	2.13E-06	6.31E-07	2.72E-05	5.08E-05	6.07E-06	1.17E-04		
RSV	1.56E-04	5.79E-04	-4.64E-05	-4.49E-05	-1.57E-05	4.55E-06	-2.20E-05	9.16E-06	2.22E-04	
picoRNA	-7.91E-04	-1.09E-03	1.46E-04	1.69E-04	1.83E-05	8.20E-07	-9.49E-05	-3.79E-05	-1.13E-04	4.14E-03

HPIV 1/3 = human parainfluenza virus (1 and 3); HPIV 2/4= human parainfluenza virus (2 and 4); HCoVs-α = human coronaviruses (229E, NL63); HCoVs-β = human coronaviruses (OC43, HKU1); picoRNA = picornaviridae virus (human rhinoviruses, enteroviruses).

Table S6. Parameters of the dynamic model.

	Influenza viruses	HCoVs-β [g]	β human coronaviruses with different R0		
R0[a]	1.7	3	2.7	5.5	8.0
T[b]	14	14	14	14	14
μ[c]	0.0714	0.0714	0.0714	0.0714	0.0714
ρ[d]	0.1214	0.2143	0.1963	0.3929	0.5714
N[e]	10000001	10000001	10000001	10000001	10000001
S[f]	10000000	10000000	10000000	10000000	10000000
I	1	1	1	1	1
R	0	0	0	0	0

[a] R0 is the average number of people infected with an infectious disease who can spread the disease to other people without external intervention and without immunity.

[b] T is the average time an infected person carries the virus.

[c] μ is the daily cure rate.

[d] ρ is the daily transmission rate.

[e] N represents the total number of people during the study period, including susceptible, infectious and recovered persons.

[f] S represents the number of susceptible people, I for infectious and R for recovered persons.

[g] HCoVs-β including human coronaviruses OC43 and HKU1.

Fig. S1. The wavelet power spectrum of respiratory viruses from 2005 to 2015. In each panel, the periodicity was showed over time (left) and average periodicity (right). Power increases from blue to red so that red indicates stronger periodicity. Black lines highlight periodicity reaching statistical significance (here, 1-year periodicity for IFV-A, Adv, EVs and hMPV). Shaded areas indicate the presence of edge effects.

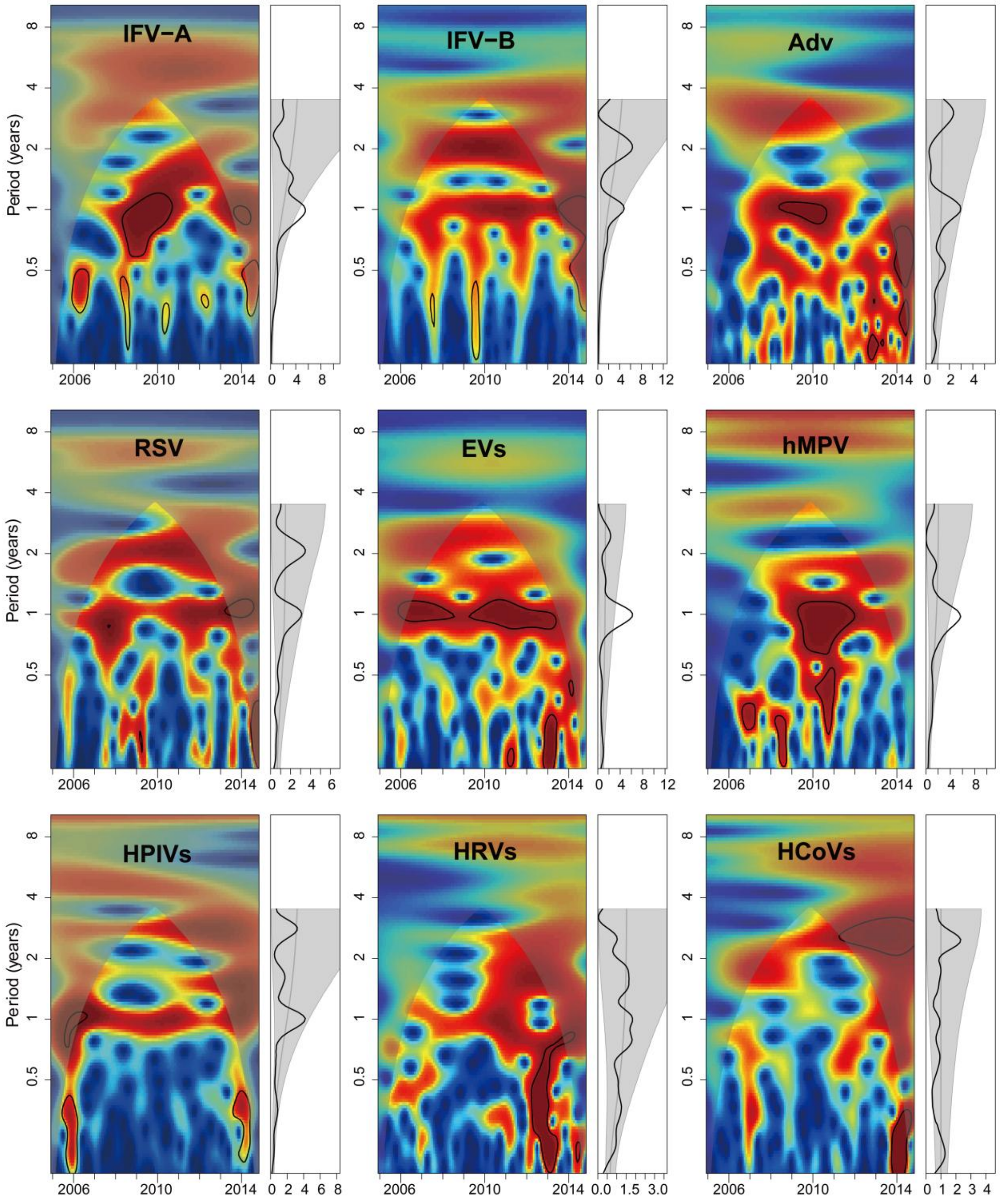

Fig. S2. Correlation coefficients of respiratory viruses, the distribution, specific value and statistical test results. The correlation coefficient greater than 0 indicates positive correlation between the two viruses, while the correlation coefficient less than 0 indicates negative correlation between the two viruses, and the correlation coefficient equal to 0 indicates no correlation between the two viruses. Statistically significant correlations are marked by asterisks. * $P<0.05$, ** $P<0.01$, *** $P<0.001$.

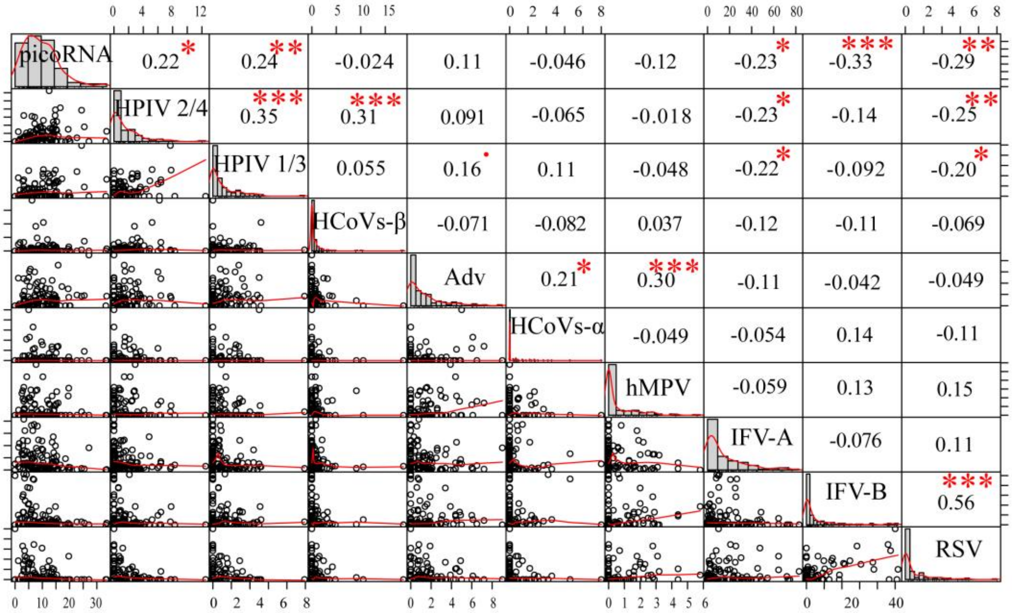

Fig. S3. The distribution of samples in each year by age group during the study period.

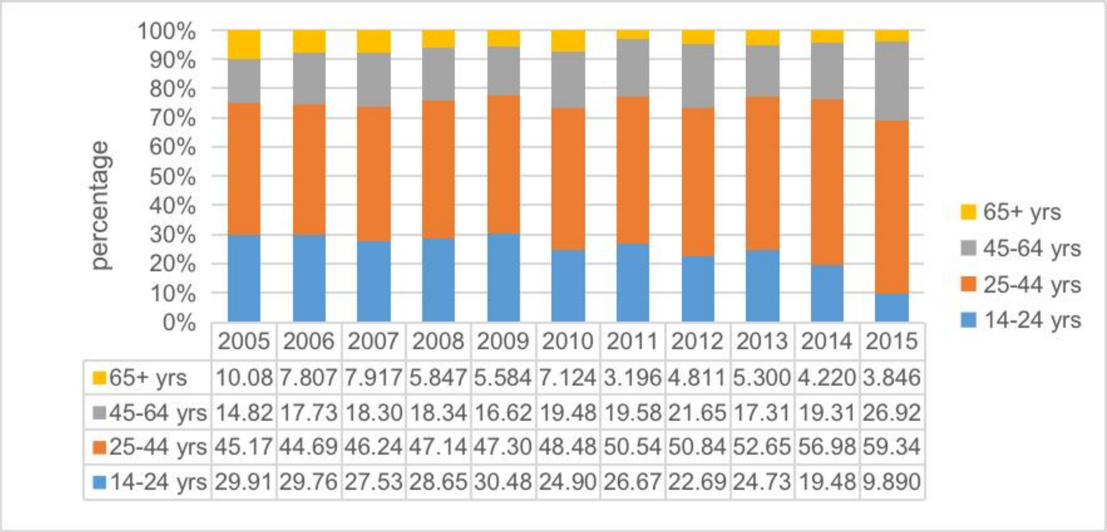

	2005	2006	2007	2008	2009	2010	2011	2012	2013	2014	2015
65+ yrs	10.08	7.807	7.917	5.847	5.584	7.124	3.196	4.811	5.300	4.220	3.846
45-64 yrs	14.82	17.73	18.30	18.34	16.62	19.48	19.58	21.65	17.31	19.31	26.92
25-44 yrs	45.17	44.69	46.24	47.14	47.30	48.48	50.54	50.84	52.65	56.98	59.34
14-24 yrs	29.91	29.76	27.53	28.65	30.48	24.90	26.67	22.69	24.73	19.48	9.890
